# Microcirculation: Current Perspective in Diagnostics, Imaging, and Clinical Applications

**DOI:** 10.3390/jcm13226762

**Published:** 2024-11-10

**Authors:** Ugur Aksu, Berna Yavuz-Aksu, Nandu Goswami

**Affiliations:** 1 Biology Department, Science Faculty, Istanbul University, Istanbul 34459, Turkey; 2 Duzen Laboratory Group, Biochemistry Section, Istanbul 34394, Turkey; bernayvz@gmail.com; 3 Gravitational Physiology and Medicine Research Unit, Division of Physiology and Pathophysiology, Otto Loewi Research Center, Medical University of Graz, 3810 Graz, Austria; 4 Center for Space and Aviation Health, Mohammed Bin Rashid University of Medicine and Health Sciences, Dubai 505055, United Arab Emirates

**Keywords:** microcirculation, hemodynamic coherence, shock, sepsis, surgery, COVID-19

## Abstract

This review discusses the pivotal role of microcirculation in maintaining tissue oxygenation and waste removal and highlights its significance in various pathological conditions. It delves into the cellular mechanisms underlying hemodynamic coherence, elucidating the roles of the endothelium, glycocalyx, and erythrocytes in sustaining microcirculatory integrity. Furthermore, the review gives comprehensive information about microcirculatory changes observed in cardiac surgery, sepsis, shock, and COVID-19 disease. Through comprehensive exploration, the review underscores the intricate relationship between microcirculation, disease states, and clinical outcomes, emphasizing the importance of understanding and monitoring microvascular dynamics in critical care settings.

## 1. Introduction

Evidence from both animal and human studies have shown that macro-hemodynamic targeted therapeutic approaches often fail to improve organ function and survival in critical situations [1,2]. In recent years, with advancements in technology particularly in the field of direct imaging, there is an increasing body of evidence that suggests that microcirculation may represent a crucial target for hemodynamic resuscitation. This represents a shift from the traditional approach of hemodynamic resuscitation which has been focused on blood pressure and cardiac output in emergency and intensive care units. This review aims to address the gap in current clinical practices where microcirculatory assessment is often overlooked despite its critical role in patient outcomes. While macro-hemodynamic targets remain central to therapeutic decision-making, they fail to fully capture the complexity of microcirculatory dysfunction, particularly in critically ill patients. By focusing on the importance of bedside microcirculatory evaluation and its potential to improve therapy control, this review seeks to emphasize the need for integrating these methods into routine clinical practice.

In conducting the literature review, we performed a comprehensive search of scientific articles using databases such as PubMed and Google Scholar. Key search terms included ‘microcirculation’, ‘organ dysfunction’, ‘COVID-19’, ‘sepsis’, ‘shock’, and ‘glycocalyx’. This search aimed to identify relevant studies, focusing on recent clinical and experimental research published up until 2024, to explore the role of microcirculatory dynamics in various pathological conditions. The literature review revealed several key trends. Recent studies highlight the diagnostic potential of microcirculatory imaging with a particular emphasis on its applications in sepsis, shock, and critical care management. Despite these advancements, significant gaps remain in routine clinical implementation, particularly due to technical and logistical barriers. This review aims to address these gaps by focusing on the current evidence supporting bedside microcirculatory assessment and its potential benefits in improving patient outcomes.

## 2. Importance of Microcirculation

The vascular components of the cardiovascular system constitute a networked structure within all organs, facilitating the transport of substances. The movement of blood through the smallest vessels of the network (vessels with a diameter of less than 100 μm, including arterioles, post-capillary venules, and capillaries) is referred to as microcirculation. The process of microcirculation permits the transfer of oxygen from the bloodstream to parenchymal cells via passive diffusion. In conditions like sepsis, microthromboses can lead to significant microvascular occlusions, further compromising oxygen delivery and exacerbating tissue hypoxia. These microvascular clots contribute to the overall disruption of microcirculatory flow, a factor that plays a critical role in the progression of sepsis-related complications. The regulation of blood flow in the tissue is dependent upon the contraction and relaxation of smooth muscles in arterioles and precapillary sphincters, which are controlled through local and neural interactions. It is therefore evident that microcirculation fulfils its primary role of meeting local metabolic needs and removing waste products from the tissues [3,4]. Oxygen is transported in the blood in two forms: bound to hemoglobin in erythrocytes (as oxyhemoglobin) and dissolved in plasma. In areas where the partial pressure of oxygen (pO2) decreases due to dissolved oxygen, hemoglobin’s affinity for oxygen is reduced, thereby facilitating the passage of oxygen through the capillary wall into the interstitium and subsequently into cells. Once inside the cell, oxygen is utilized in the electron transport chain within the inner mitochondrial membrane facilitating the production of adenosine 5′-triphosphate (ATP) through oxidative phosphorylation. It is therefore evident that microcirculation plays a pivotal role in ensuring ATP production by maintaining a consistent and adequate oxygen supply and by regulating its levels in accordance with the cellular demand [5].

“In conditions like sepsis, microthromboses can lead to significant microvascular occlusions, further compromising oxygen delivery and exacerbating tissue hypoxia. These microvascular clots contribute to the overall disruption of microcirculatory flow, a factor that plays a critical role in the progression of sepsis-related complications”.

## 3. Cellular Processes of Hemodynamic Coherence

**Figure 1:**
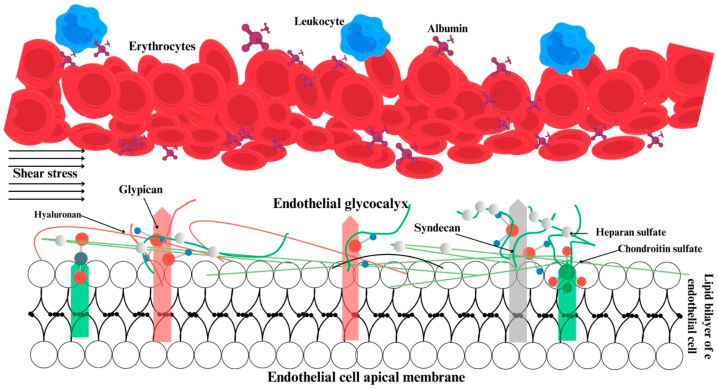
Structural and functional relations within the glycocalyx, endothelium, and erythrocytes. Green, pink and gray bars show the anchor components of glycocalyx.

The maintenance of microcirculation and its distinction from macrohemodynamics are contingent upon physiological events occurring in and around the endothelium. Three key elements are responsible for hemodynamic coherence: the glycocalyx, the endothelium, and erythrocytes (Figure 1).

The glycocalyx is defined as a reticulated layer of membrane-bound proteoglycans and glycoproteins that surrounds the lumen-facing surface of the endothelium. Additionally, the glycocalyx contains a variety of substances including antioxidants, growth factors, and anticoagulants, which are collected from plasma for use when necessary [6,7]. In physiological conditions, there is a state of equilibrium between the processes of glycocalyx synthesis and degradation. The degradation of the glycocalyx is facilitated by inflammatory processes, whereas optimal tissue perfusion is ensured by glycocalyx synthesis, which is promoted by shear stress [8,9]. The negatively charged glycocalyx structure of the cell surface prevents blood and endothelial cells from coming into contact with one another [6]. Damage to the glycocalyx results in a reduction in the distance between erythrocytes and the endothelium, leading to disruption of microvascular perfusion [10]. Furthermore, the glycocalyx plays a role in regulating the adhesion of leukocytes and erythrocytes to each other and to the endothelium [6,11] (Figure 1).

Secondly, the endothelium itself, its junctional complexes, and the elements of the glycocalyx act as a selectively permeable barrier between the inside and outside of the vessel. This barrier permits the retention of plasma and large molecular-weight protein structures within the vessel [12]. The regulation of capillary wall permeability is specifically provided by glycosaminoglycans, sialic acid, and proteins gathered from plasma within the glycocalyx structure [6,13]. While studies indicate that glycocalyx disruption may result in capillary leakage and that the extent of the leakage is associated with the quantity of the components shed from the glycocalyx [14,15,16], recent studies suggest that the glycocalyx itself does not contribute to capillary permeability [17,18].

Furthermore, the glycocalyx serves to convey shear stress data generated by the erythrocytes to the endothelium, thereby regulating vascular tone and endothelial function [19]. Impairment of the glycocalyx has been demonstrated in numerous pathological conditions, including sepsis, septic shock, cardiogenic shock, and coronavirus disease, as well as in surgical procedures such as cardiopulmonary bypasses and transplantations. This is associated with a poor prognosis. In addition to their pathophysiological significance, conditions such as sepsis, septic shock, cardiogenic shock, and COVID-19 have a profound global impact. Sepsis alone accounts for millions of deaths worldwide annually, representing a significant burden on healthcare systems, particularly in low- and middle-income countries. The global spread of COVID-19 has further highlighted the need for effective microcirculatory management in critical care as well as the long-term economic and public health implications of such conditions [20,21,22,23,24,25].

The endothelial layer in the arterioles regulates microcirculation through the release of mediators that induce vascular contraction and relaxation, a process that occurs independently of the glycocalyx. This function is fulfilled through autocrine and paracrine interactions [26,27]. The endothelium not only processes local hemodynamic information but also plays a pivotal role in regulating hemostasis, ensuring unimpeded blood flows within the vessel. The process of hemostasis is regulated by molecules released from the endothelium, including von Willebrand factor (vWF), tissue factor (TF), and plasminogen activator inhibitor type 1 (PAI-1). During the period of inflammation, the balance between prothrombotic and antifibrinolytic processes can lead to microvascular occlusion, resulting in ischemia and subsequent multiple organ dysfunction syndrome (MODS) [26,28].

Proinflammatory cytokines play a pivotal role in the meditation of systemic inflammation. The endothelium responds to these cytokines in order to regulate its barrier function, vascular tone, and coagulation process. When activated by proinflammatory cytokines, the endothelial barrier is compromised, and the endothelium synthesizes specific molecules on its luminal surface, facilitating the adhesion of leukocytes and erythrocytes [26,29]. At this juncture, there is a notable degree of interdependence between the glycocalyx and the endothelium [6,7]. The weakened barrier results in tissue oedema, and the adherence of leukocytes to the endothelium causes obstructions in the microvascular beds [28]. The presence of oedema and obstructions in microcirculation can be readily identified using imaging devices [30,31].

Lastly, erythrocytes, which have a diameter of approximately 7–8 μm in the absence of external forces, must undergo a deformation of their membranes in order to traverse capillaries during movement. This reversible deformation entails a transformation in geometric shape without any alteration in surface area, thereby enabling erythrocytes to traverse through capillaries with a diameter of 3–5 μm [32]. A reduction in erythrocyte deformability has been demonstrated in numerous pathologies, including sepsis. A reduction in deformability results in an increase in blood viscosity. The stiffening of cells impedes microvascular flow, and oxygenation is further impaired by hemoglobin’s reduced affinity for oxygen [33]. The deformability of individual erythrocytes can be quantified using techniques such as micropipette aspiration, atomic force microscopy, and optical trapping. The deformability of groups of erythrocytes can be assessed using methods including quantitative phase imaging, filtration, microfluidic filtration, and laser diffractometry [34]. A reduction in erythrocyte deformability has been linked to a decline in capillary density and survival in sepsis [35,36].

A number of processes can occur concurrently in critical illness, including a systemic inflammatory response, damage to the glycocalyx, endothelial dysfunction, endothelial activation, coagulation processes, and erythrocyte deformability problems. These processes, which occur in and around the endothelium, disrupt the relationship between the macro- and microenvironments, leading to a loss of coherence. This relationship underscores the importance of maintaining microcirculatory function to improve critical illness outcomes and highlights why targeting microcirculation could be transformative in clinical practice. Hence, the extent to which current therapeutic approaches can improve microcirculation through cellular processes remains a matter of debate.

## 4. Importance of Microcirculation in Shock

The term “shock” can be defined as the inadequate supply and utilization of oxygen in tissues, which significantly impacts microcirculation. Although hypotension is a crucial indicator of shock, the activation of the sympathetic nervous system often results in a masking of a drop in blood pressure. Furthermore, while the lower limit for systolic pressure is typically regarded as 90 mmHg, lower values may be considered normal due to inter-individual variability. Four distinct categories of shock have been identified: hypovolemic, cardiogenic, obstructive, and distributive shock. In hypovolemic, cardiogenic, and obstructive shock, cardiac output is low, and the convection characteristics of microcirculation are weak. Conversely, in distributive shock, high cardiac output is typically observed in the early stages, with microcirculation being redirected by inflammatory-oxidant mediators. It is possible for multiple types of shock to occur simultaneously, such as distributive-cardiogenic and hypovolemic-cardiogenic [37].

The primary objective of resuscitation in all four types of shock is to enhance tissue perfusion and oxygenation. Macro-hemodynamic variables, such as blood pressure, are managed within a specific range with the assumption that this will improve hypoxia and hypoperfusion. Nevertheless, research has demonstrated that pursuing macro-hemodynamic objectives does not invariably enhance oxygenation [38], organ performance [2], or survival [39,40,41]. The damage to organs and fatalities observed during and after shock treatment are attributed to hemodynamic incoherence. In physiological conditions, improvements in microcirculation follow improvements in systemic hemodynamics, indicating a synergy between macro- and microcirculation. This is known as the haemodynamic coherence concept, and it leads to a correction of the shock [42]. However, it has been demonstrated that microcirculatory improvement does not always follow systemic hemodynamic improvement [43]. The loss of hemodynamic coherence has been shown to impair oxygenation by reducing tissue oxygen extraction. It has been established that hemodynamic incoherence is directly associated with four different types of microcirculatory changes, all of which can be monitored using direct imaging systems. These changes have been observed to uniformly show a decrease in capillary density and oxygen-carrying capacity.

Type 1: Sepsis is an example of a Type 1 alteration. In this type, there is a discrepancy in the flow of blood between different capillaries. Furthermore, even in vessels with a flow, there is a disparity in flow rate between them. This heterogeneous vascular flow exceeds the physiological limits, thereby affecting both the convection and diffusion characteristics of microcirculation. Consequently, the density of capillaries and the flow of microvasculature are both impaired in Type 1 alterations.

Type 2: The second type of alteration is observed in conditions such as fluid overload, which frequently occurs during bypass surgery. As a consequence of dilution, both the number of erythrocytes per unit blood volume and the space between them decrease. The diffusion characteristics of microcirculation are particularly affected.

Type 3: Vasoactive agents, such as noradrenaline, or increased venous pressure may result in a reduction or cessation of microvascular flow. In this type of change, the convection characteristics of microcirculation are affected.

Type 4: This type occurs when capillary leakage causes tissue oedema. Oedema can increase the diffusion distance between erythrocytes and cells, thereby affecting oxygen extraction (Table 1) [44].

## 5. Microcirculatory Changes in Sepsis

The term “sepsis” is used to describe organ dysfunction resulting from an uncontrolled host response to infection. If not promptly identified and treated, it can result in septic shock, multi-organ failure, and mortality. Despite the administration of fluids in cases of septic shock, hypotension (defined as a mean arterial pressure of less than 65 mmHg) and perfusion disorder (defined as a lactate level exceeding 2 mmol/L) remain uncorrected [45].

The microcirculation of patients with sepsis differs significantly from that of healthy individuals and even non-septic ICU patients, with a notable redirection of microcirculatory flow [46]. Leukocyte and platelet-induced obstructions resulting from endothelial dysfunction lead to the redirection of blood flow. The increased diffusion distance caused by shunted microcirculation reduces oxygen consumption. In septic conditions, microthromboses by platelets play a critical role in microcirculatory shunting, leading to impaired oxygen delivery and contributing to organ dysfunction. These microthromboses are often overlooked but are significant contributors to the pathophysiology of sepsis. Moreover, oxygen extraction is increased in capillaries that remain perfused, but not uniformly across microcirculation. This imbalance often results in elevated central venous oxygen saturation, as a significant proportion of cardiac output is shunted through microcirculatory regions where no effective oxygen extraction occurs [47].

Decreased perfusing capillaries and increased flow heterogeneity are hallmarks of sepsis. These changes are more pronounced in non-survivors than in survivors and improve over time in survivors. Changes in microcirculation are independent predictors of mortality in septic patients [48].

The phenomenon of microcirculatory heterogeneity permits oxygen to gain access to the venous system prior to utilization, which in turn affects the extraction of oxygen from tissues. In addition to microcirculatory heterogeneity, decreased functional density and slowed erythrocyte velocity are observed in sepsis [49].

While erythrocyte velocity may be similar in survivors and non-survivors, perfused capillary density and heterogeneity are prominent in non-survivors. The variables determining oxygen diffusion are more related to the sepsis picture than erythrocyte velocity, which directly determines convection [50].

The inflammatory process and systemic vasodilation caused by mediators released in sepsis create a hypovolemic-like situation [51]. Although fluid resuscitation is the initial step in improving macro-hemodynamic variables in sepsis and septic shock, there is still debate regarding the optimal type of fluid and the appropriate volume to administer. Furthermore, disturbances in microcirculation and an inadequate response to resuscitation are directly correlated to survival outcomes. Nevertheless, fluid administration can increase blood flow by enhancing cardiac output and thus the propulsive power of the blood, thereby fulfilling the convective component of oxygen transport. Conversely, the administration of substantial quantities of fluid may result in the elevation of oxidant mediators within the vessel, leading to the disruption of endothelial integrity and damage of surface elements. In this context, oedema may ensue as intravascular fluid leaks into the interstitial space. The formation of oedema can be attributed to either the direct effect of the fluid or the disruption of cell–cell connections and surface elements, which is a consequence of the nature of sepsis itself. The presence of oedema may result in a reduction in blood flow as an indication of high level of lactate due to the creation of pressure within the perivascular space and an increase in the distance between capillaries and cells. The development of edema can compress microcirculatory vessels, thereby increasing reliance on anaerobic glycolysis, which is clinically detectable through elevated lactate levels. This accumulation of lactate serves as an indicator of inadequate oxygenation and impaired microcirculatory function in critically ill patients. This can lead to a limitation in the efficiency of gas exchange. Consequently, both convection and diffusion are impaired but macrohemodynamics may remain within acceptable physiological limits, a condition known as hemodynamic incoherence [52,53].

## 6. Microcirculatory Changes in Cardiac Surgery

Cardiopulmonary bypass surgery has a significant impact on the body’s hemodynamic and biochemical processes. During extracorporeal circulation, systemic hemodynamics are maintained within desired ranges, although there is a decrease in oxygen delivery and hemoglobin concentration. Additionally, the density of perfused capillaries decreases. These alterations in microcirculation are accompanied by an increase in hemoglobin oxygen saturation and erythrocyte velocity at the capillary level, resulting from impaired oxygen extraction [34]. The administration of blood transfusions following surgical procedures has been demonstrated to enhance capillary density and hemoglobin levels in the absence of an increase in microvascular flow. This occurs independently of hemodynamics and volume status, thereby improving oxygen delivery [35]. Furthermore, reducing the size of the external pump may also prevent microvascular hypoperfusion [35]. However, the pulsatile nature of the pump [54] and the composition of the inner lining material of the cannula circuit [55] do not influence microvascular capillary density or perfusion impairment during surgical procedures.

The contact of blood with a foreign surface during cardiopulmonary bypass surgery induces a systemic inflammatory response (SIRS), affecting all levels from systemic to subcellular [56,57]. The presence of elevated cytokine levels in the blood and visualization of leukocytes adhering directly to the endothelium [58] and microaggregates in capillaries [59] serve to illustrate the inflammatory process. The presence of microaggregates in the postoperative period may be an indicator of an increased risk of stroke [59].

Cardiac surgery can be performed without using a cardiopulmonary bypass circuit, which has the effect of reducing cardiac output directly. A reduction in cardiac output affects microvascular flow, impacting all variables related to convection. While pump use has been shown to decrease microvascular hematocrit and blood viscosity, these variables remain unaffected in non-pump surgery [34,60].

## 7. Microcirculatory Changes in Coronavirus Disease 2019 (COVID-19)

**Figure 2:**
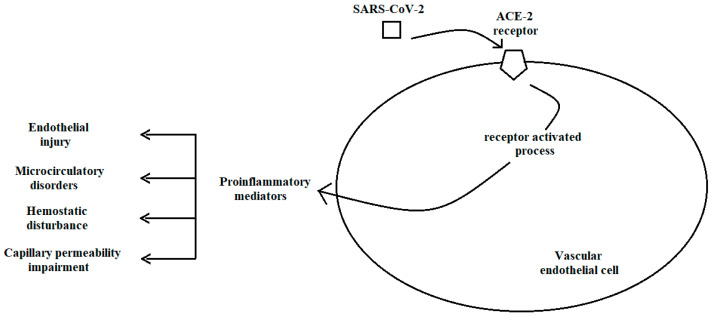
Effects of severe acute respiratory syndrome coronavirus 2 (SARS-CoV-2) on vascular endothelial cells (ACE-2 receptor: Angiotensin converting enzyme-2 receptor).

Severe acute respiratory syndrome coronavirus 2 (SARS-CoV-2) is the strain of the coronavirus responsible for the respiratory illness known as coronavirus disease 2019 (COVID-19) which triggered the global pandemic. The initial identification of the virus in Wuhan, China, was followed by a rapid global dissemination [61]. Given its propensity for transmission, SARS-CoV-2 gains entry to vascular endothelial cells via the angiotensin-converting enzyme (ACE) 2 receptor [62]. Activation of the ACE-2 receptor results in a significant release of pro-inflammatory mediators, collectively termed “cytokine storms”, which contribute to endothelial damage [63]. Endothelial dysfunction presents in a variety of ways, including impaired capillary permeability and disturbances in hemostasis [64] (Figure 2).

The presence of microvascular thrombosis is a prominent feature observed in microcirculatory images of patients diagnosed with COVID-19 [65]. It is possible that this thrombosis contributes to the underlying oxygen delivery deficiencies observed in patients with COVID-19. The resolution of thrombosis is a prerequisite for the management of conditions associated with SARS-CoV-2, such as acute respiratory distress syndrome (ARDS) and myocardial damage [63,66].

In patients with COVID-19, despite blood pressure values within the normal ranges, there may be a high heart rate, slightly elevated lactate levels, low systemic hemoglobin, and decreased hematocrit values [67]. It is noteworthy that perfused vessel density, erythrocyte velocity, and capillary hematocrit levels are elevated in the microcirculation of these patients. The elevated capillary hematocrit results from capillary plasma leakage due to vascular barrier dysfunction caused by endothelial and glycocalyx damage, despite a reduction in systemic hematocrit [68].

The microcirculatory characteristics observed in patients with coronavirus disease 2019 (COVID-19) differ from those observed in patients with sepsis. While microcirculation in sepsis and septic shock typically exhibits heterogeneity with reduced vascular density and impaired flow [46,69], patients with coronavirus disease 2019 (COVID-19) display a non-heterogeneous microcirculation [70]. Furthermore, reductions in functional capillary density may not be observed in patients with COVID-19 [48,71,72]. SARS-CoV-2 differs from bacterial sepsis but is still considered a form of viral sepsis disease [73].

The maintenance of functional capillaries in patients with COVID-19 represents a compensatory mechanism that increases oxygen extraction in response to hypoxia induced by hyperinflammation and hypercoagulation [67]. Nevertheless, in patients with severe SARS-CoV-2 infection and high sequential organ failure assessment (SOFA) scores, characterized by increased leukocytes and microaggregates in microcirculation, this compensatory mechanism may prove ineffective [67]. Mechanical ventilation has been observed to increase erythrocyte velocity in patients with COVID-19 [72]. However, the impact of mechanical ventilation on diffusion distance against increased erythrocyte velocity remains unknown. Additionally, various studies have reported high vascular densities in the sublingual microcirculation of patients with confirmed or suspected cases of SARS-CoV-2 infection, alongside decreases in perfused vessels and flow velocity [74]. Notwithstanding the absence of a global pandemic caused by the SARS-CoV-2 virus, comprehensive and large-scale studies of microcirculatory changes in patients diagnosed with the disease will facilitate a deeper understanding of the nature of viral pandemics derived from SARS-CoV-2 and inform the development of more effective prevention and treatment strategies in the future.

## 8. Assessment and Visualization of Microcirculation

The intricate processes occurring in microcirculation are currently only elucidated at the research level, employing imaging techniques that are not yet integrated into routine clinical practice (Table 2). Nevertheless, to some extent, the microenvironment is in fact assessed in the clinical setting. For instance, the color of the skin can provide some indication of the state of microcirculation in the skin. In addition to color changes, the skin blood supply can be estimated by measuring temperature or capillary refill time. A reduction in skin temperature in surgical patients has been linked to low cardiac output, low central venous oxygen saturation, and elevated lactate levels [75]. The measurement of capillary refill time is a straightforward method that has been linked to blood lactate levels, the sequential organ failure assessment (SOFA) score, and survival in patients with sepsis. However, inter-clinician variability in assessment could potentially impact the results. Despite impaired skin microcirculation in conditions such as hypertension and renal failure, the relationship between skin and systemic circulation remains poorly understood [76,77].

The measurement of blood lactate levels represents a widely utilized biochemical approach for the evaluation of disruptions in microcirculation. Besides the requirement of vasopressor to maintain a mean arterial pressure of 65 mmHg, lactate levels above 2 mM indicate the presence of shock, and even minor fluctuations in lactate levels are associated with direct correlations to survival rates [78]. It should be noted that although lactate is a product of anaerobic metabolism, exogenous catecholamines or decreased hepatic clearance may also cause an increase in lactate levels [79]. Moreover, lactate levels might not increase even if oxygenation is poor and blood pressure is within normal limits [1]. Therefore, it cannot be assumed that evaluating microcirculation based solely on blood lactate levels is a reliable method.

Near-infrared spectroscopy (NIRS) devices are capable of measuring oxygenation at the microcirculatory level. However, it is important to note that the technique has significant limitations when it comes to evaluating microcirculation. A significant challenge is the inability of these devices to provide the requisite detailed spatial resolution for the accurate assessment of microvascular structures. NIRS primarily measures changes in oxygenated and deoxygenated hemoglobin within capillaries, arterioles, and venules. Furthermore, NIRS signals are frequently affected by the absorption and scattering of light in diverse tissue layers, rendering it challenging to discern the precise impact of the microcirculatory blood flow from the overall tissue oxygenation. This limitation is particularly problematic in heterogeneous tissues where multiple layers of blood vessels and different tissue types may contribute to the signal, thereby complicating the interpretation of the microcirculatory dynamics. Furthermore, NIRS does not provide direct information on red blood cell velocity, capillary density, or microvascular perfusion heterogeneity, which are crucial parameters for a comprehensive assessment of microcirculation [80,81].

Retinal vessel diameter (RVD) measurement is a technique used to evaluate the diameters of arteries and veins in the retina. While it provides valuable information about the structure and function of the retinal vasculature, RVD has also significant limitations when it comes to assessing microcirculation. Firstly, RVD measurements typically focus on larger and medium-sized vessels which do not provide direct information about microcirculation’s smallest components, such as capillaries. Therefore, RVD measurements do not adequately reflect changes or dysfunctions in microcirculation. Additionally, RVD measurements are designed to assess structural and geometric changes in retinal arterioles and venules, but they do not measure the functional dynamics of microcirculation such as blood flow rates, oxygenation status, or microvascular reactivity. Additionally, RVD measurements lack the ability to distinguish whether observed changes occur at the microcirculatory level or in the larger vascular network. For example, observed changes such as arteriolar narrowing or venular dilation may not be a direct result of pathological processes occurring in microcirculation, potentially leading to misleading conclusions about the state of microcirculation [82,83].

A variety of imaging devices can be employed to ascertain information regarding tissue perfusion. Laser speckle contrast and photoacoustic imaging systems represent non-invasive imaging techniques. The acquisition of real-time images is achieved through the alteration of the optical properties of the transmitted light subsequent to the excitation of the tissue or by the absorption of light by the tissue. While these devices may be suitable for skin imaging in terms of ergonomics, they are not practical for other parts of the body [84,85]. Additionally, the combination of laser-Doppler flowmetry with white light laser technology provides an enhanced assessment of microcirculatory oxygen uptake, allowing for a more comprehensive evaluation of tissue perfusion and oxygenation. This approach is valuable in detecting subtle changes in microvascular function that may not be visible through single-modality techniques. Furthermore, these devices provide information about the convection properties of microcirculation, which is a significant factor influencing oxygen transport. However, these techniques are non-quantitative, provide only percentage change information, are affected by individual differences, and, more importantly, are unable to provide diffusion-related information in microcirculation. The transport of oxygen to the tissue is determined by two processes: convection and diffusion. In other words, for the tissue to receive oxygen, it must pass at a certain speed from a location in close proximity to the cell. It is therefore evident that in order to evaluate the oxygen transport capacity of microcirculation, both microvascular flow (convection) and capillary distance (diffusion) information are required. In order to achieve this, it is necessary to conduct direct visualization of microcirculation [3].

The direct imaging of microcirculation without the use of any dye has been a viable technique in clinical research for the past 25 years. For the first time, microcirculation was imaged at the bedside in humans using orthogonal polarization spectral (OPS) technology. Subsequently, technological advancements led to the development of two subsequent generations of devices using dark field microscopy: initially the sidestream dark field (SDF) and subsequently the incident dark field (IDF) approach. All three generations of devices are based on the principle that hemoglobin becomes visible by absorbing green light. In the images, microvessels can be easily visualized, and the velocity of small vessels and their quantity in the region of interest can be determined. Consequently, the second component of oxygen transport, namely diffusion information, can also be obtained [86,87].

The sublingual region has typically been evaluated with imaging devices. Microvessels are identified according to their flow and branching direction. Despite the sublingual region’s distance from the heart and other organs, it is a well-defined area for understanding pathophysiology and treatment efficacy [49,88]. In addition to the sublingual region, other areas have been imaged, including the vagina [89], colon [90], skin [91], rectum [92], labia [93], conjunctiva [94], brain [95], and peritoneum [96].

A novel system has recently been developed for the simultaneous assessment of microvascular oxygen saturation and microcirculatory variables. This system maps oxygen saturation in microcirculation based on the principle that oxyhemoglobin absorbs blue light. It has been demonstrated that there is a correlation between the inspired oxygen fraction (FiO_2_) and microvascular saturation and that local saturation changes were associated with changes in the microcirculation [97].

**Table 2:** An overview of assessment and visualization of microcirculation.

Method	Advantages	Limitations	Typical Applications
Sublingual Microscopy (OPS, SDF, and IDF)	Non-invasive, real-time visualization of microcirculation; Direct visualization of microvessels	Limited to surface areas like sublingual region; Requires special equipment and training	Bedside monitoring in ICU, sepsis, critical care, microvascular research; Assessment of microcirculatory dynamics in critically ill patients
Laser-Doppler Flowmetry (LDF)	Continuous measurement of blood flow	No structural information	Research; Experimental settings
Near-Infrared Spectroscopy (NIRS)	Non-invasive, easy to apply; Indirect measure of tissue oxygenation	Indirect measure of tissue oxygenation	Tissue oxygenation in trauma; Surgery
Retinal Vessel Diameter (RVD) Measurement	Provides data on retinal vasculature	Does not provide direct information on microcirculation	Retinal vessel assessment; Cardiovascular risk evaluation [98,99,100,101,102,103,104]
Laser Speckle Contrast Imaging	Non-invasive, real-time imaging of microcirculation	Non-quantitative, provides only percentage change data	Skin imaging; Microcirculation monitoring in research settings
Photoacoustic Imaging	Non-invasive, detailed microvascular imaging	Limited to specific tissue areas	Skin and tissue perfusion monitoring

## 9. Variables Obtained from Microcirculation Images

The rise in sublingual microcirculation studies has enabled the acquisition of a multitude of variables. A consensus was reached at meeting in 2007 and 2018 regarding the variables related to capillary density, perfusion, and erythrocyte flow behavior [105,106]. While the initial approach was predominantly based on manual measurements, more recently, novel variables have been proposed that were calculated using computer software [107]. These variables in question are now outlined as follows.

Microvascular Flow Index (MFI): The MFI provides information regarding the quality of perfusion in the region of interest. The video image is hypothetically divided into four parts, with each part assigned a value between 0 and 3 according to the observed flow type. The MFI value is presented as the mean without units (with 0 indicating no flow; 1 indicating intermittent flow; 2 indicating slow flow; 3 indicating continuous flow).

Total Vessel Density (mm/mm²) (TVD): The TVD is calculated as the total length of vessels divided by the total surface area of the region of interest. This provides information about the vessel density in the region.

Perfused Vessel Density (mm/mm²) (FCD): The FCD is calculated as the total perfused vessel length divided by the total surface area. This variable provides information about functional vessel density.

Proportion of Perfused Vessels (%) (PPV): The PPV is calculated as the number of perfused vessels per hundred divided by the total number of vessels and provides information about perfusion quality.

Flow Heterogeneity Index (FHI): This provides information about perfusion heterogeneity. The calculation is performed by dividing the difference between the highest flow and the lowest flow indices by the mean microvascular flow index [105,106,107].

## 10. Conclusions

The direct visualization of microcirculation without the use of dyes has been possible for approximately 25 years within the advent of handheld microcirculatory imaging devices. In recent years, there has been a notable surge in interest in microcirculation, which has become a subject of investigation across various disciplines. The capacity to directly observe and quantify microcirculatory function at the bedside with the aid of handheld vital microscopes enables the translation of findings from basic research, ultimately benefiting patients. Furthermore, given that the shortcomings of macro-hemodynamic targets remain a topic of contention, the monitoring of microcirculation is of particular importance in order to minimize fluid administration and to avoid the use of unnecessary medications.

In other words, when a microcirculation-guided therapeutic approach addresses the root causes of critical illnesses, physicians will be able to discern how endothelial and microvascular dysfunction not only contribute to organ damage but also impede treatment strategies in complex illness conditions.

Sublingual microscopy is currently a valuable tool for bedside microcirculatory assessment. However, other techniques, including laser-Doppler flowmetry and advanced imaging systems, also offer promising avenues for future therapeutic monitoring. Each of these methods can and will play a role in enhancing therapy control by providing detailed insights into microcirculatory dynamics. The future integration of these diverse methods in clinical practice will provide significant improvements in patient outcomes.

Despite the growing use of sublingual microscopy and other microcirculatory assessment tools, there remains debate over their clinical utility in different conditions, such as sepsis and COVID-19. Conflicting findings regarding the benefits of microcirculation-guided therapy have raised questions about the optimal therapeutic targets. Furthermore, while some studies suggest a strong correlation between microcirculatory improvements and patient outcomes, others have not observed significant clinical benefit, particularly in the context of fluid resuscitation in sepsis. These controversies underscore the need for further research to determine the most effective application of microcirculatory monitoring in clinical practice.

However, the situation is somewhat different in the real world. Despite the considerable interest in microcirculation, the imaging of microcirculation and the evaluation of events remain outside the scope of routine clinical practice. The primary reason for this is that the data obtained from imaging techniques exclusively pertains to the oxygen-carrying capacity of the blood at the tissue level. However, the most significant shortcoming is the lack of assessment of the actual oxygen saturation levels in the blood that reach the cells through microcirculation and the cellular energy metabolism in this region. In broader terms, the viability of cells can be evaluated by determining the proportion of oxygen delivered to the patient that is delivered to the cells and the ability of these cells to utilize the oxygen supplied. The development of microcirculation imaging technologies in this direction may enable a reassessment of traditional treatment methods in pathological conditions related to emergency medicine, intensive care, surgery, and internal medicine.

## Figures and Tables

**Table 1:** An overview of microcirculatory changes.

Change Type	Example Condition	Characteristics	Affected Aspects
Type 1	Sepsis	Discrepancy in flow of blood between different capillaries, heterogeneous microvascular flow exceeding physiological limits	Capillary density, convection and diffusion characteristics
Type 2	Fluid overload	Decrease in the number of erythrocytes per unit blood volume and space between them due to dilution	Diffusion characteristics of microcirculation
Type 3	Vasoactive agents (e.g., norepinephrine)	Reduction or cessation of microvascular flow due to vasoactive agents or increased venous pressure	Convection characteristics of microcirculation
Type 4	Capillary leakage causing tissue edema	Increased diffusion distance between erythrocytes and cells due to edema	Diffusion characteristics of microcirculation and oxygen extraction efficiency

## References

[1] Aksu U., Bezemer R., Yavuz B., Kandil A., Demirci C., Ince C. (2012). Balanced vs unbalanced crystalloid resuscitation in a near-fatal model of hemorrhagic shock and the effects on renal oxygenation, oxidative stress, and inflammation. Resuscitation.

[2] den Uil C.A., Lagrand W.K., van der Ent M., Nieman K., Struijs A., Jewbali L.S., Constantinescu A.A., Spronk P.E., Simoons M.L. (2014). Conventional hemodynamic resuscitation may fail to optimize tissue perfusion: An observational study on the effects of dobutamine, enoximone, and norepinephrine in patients with acute myocardial infarction complicated by cardiogenic shock. PLoS ONE.

[3] Guven G., Hilty M.P., Ince C. (2020). Microcirculation: Physiology, Pathophysiology, and Clinical Application. Blood Purif..

[4] Klijn E., Den Uil C.A., Bakker J., Ince C. (2008). The heterogeneity of the microcirculation in critical Illness. Clin. Chest Med..

[5] Mik E.G. (2013). Special article: Measuring mitochondrial oxygen tension: From basic principles to application in humans. Anesth. Analg..

[6] Villalba N., Baby S., Yuan S.Y. (2021). The Endothelial glycocalyx as a double-edged sword in microvascular homeostasis and pathogenesis. Front. Cell Dev. Biol..

[7] Kolářová H., Ambrůzová B., Svihálková Šindlerová L., Klinke A., Kubala L. (2014). Modulation of endothelial glycocalyx structure under inflammatory conditions. Mediat. Inflamm..

[8] Arisaka T., Mitsumata M., Kawasumi M., Tohjima T., Hirose S., Yoshida Y. (1995). Effects of shear stress on glycosaminoglycan synthesis in vascular endothelial cells. Ann. N. Y. Acad. Sci..

[9] Mulivor A.W., Lipowsky H.H. (2004). Inflammation- and ischemia-induced shedding of venular glycocalyx. Am. J. Physiol. Heart Circ. Physiol..

[10] Lee D.H., Dane M.J., van den Berg B.M., Boels M.G., van Teeffelen J.W., de Mutsert R., den Heijer M., Rosendaal F.R., van der Vlag J., van Zonneveld A.J. (2014). Deeper penetration of erythrocytes into the endothelial glycocalyx is associated with impaired microvascular perfusion. PLoS ONE.

[11] Nishiguchi E., Okubo K., Nakamura S. (1998). Adhesion of human red blood cells and surface charge of the membrane. Cell Struct. Funct..

[12] Myburgh J.A., Mythen M.G. (2013). Resuscitation fluids. N. Engl. J. Med..

[13] Cioffi D.L., Pandey S., Alvarez D.F., Cioffi E.A. (2012). Terminal sialic acids are an important determinant of pulmonary endothelial barrier integrity. Am. J. Physiol. Lung Cell. Mol. Physiol..

[14] Tang T.H., Alonso S., Ng L.F., Thein T.L., Pang V.J., Leo Y.S., Lye D.C., Yeo T.W. (2017). Increased serum hyaluronic acid and heparan sulfate in dengue fever: Association with plasma leakage and disease severity. Sci. Rep..

[15] Suwarto S., Sasmono R.T., Sinto R., Ibrahim E., Suryamin M. (2017). Association of endothelial glycocalyx and tight and adherens junctions with severity of plasma leakage in dengue infection. J. Infect. Dis..

[16] Lam P.K., McBride A., Le D.H.T., Huynh T.T., Vink H., Wills B., Yacoub S. (2020). Visual and biochemical evidence of glycocalyx disruption in human dengue infection, and association with plasma leakage severity. Front. Med..

[17] Guerci P., Ergin B., Uz Z., Ince Y., Westphal M., Heger M., Ince C. (2019). Glycocalyx degradation is independent of vascular barrier permeability increase in nontraumatic hemorrhagic shock in rats. Anesth. Analg..

[18] Ergin B., Guerci P., Uz Z., Westphal M., Ince Y., Hilty M., Ince C. (2020). Hemodilution causes glycocalyx shedding without affecting vascular endothelial barrier permeability in rats. J. Clin. Transl. Res..

[19] Mochizuki S., Vink H., Hiramatsu O., Kajita T., Shigeto F., Spaan J.A., Kajiya F. (2003). Role of hyaluronic acid glycosaminoglycans in shear-induced endothelium-derived nitric oxide release. Am. J. Physiol. Heart Circ. Physiol..

[20] Saoraya J., Wongsamita L., Srisawat N., Musikatavorn K. (2021). Plasma syndecan-1 is associated with fluid requirements and clinical outcomes in emergency department patients with sepsis. Am. J. Emerg. Med..

[21] Schmidt E.P., Overdier K.H., Sun X., Lin L., Liu X., Yang Y., Ammons L.A., Hiller T.D., Suflita M.A., Yu Y. (2016). Urinary glycosaminoglycans predict outcomes in septic shock and acute respiratory distress syndrome. Am. J. Respir. Crit. Care Med..

[22] Jung C., Fuernau G., Muench P., Desch S., Eitel I., Schuler G., Adams V., Figulla H.R., Thiele H. (2015). Impairment of the endothelial glycocalyx in cardiogenic shock and its prognostic relevance. Shock.

[23] Yamaoka-Tojo M. (2020). Endothelial glycocalyx damage as a systemic inflammatory microvascular endotheliopathy in COVID-19. Biomed. J..

[24] Akande O.W., Akande T.M. (2020). COVID-19 pandemic: A global health burden. Niger. Postgrad. Med. J..

[25] La Via L., Sangiorgio G., Stefani S., Marino A., Nunnari G., Cocuzza S., La Mantia I., Cacopardo B., Stracquadanio S., Spampinato S. (2024). The Global Burden of Sepsis and Septic Shock. Epidemiologia.

[26] Galley H.F., Webster N.R. (2004). Physiology of the endothelium. Br. J. Anaesth..

[27] Juffermans N.P., van den Brom C.E., Kleinveld D.J.B. (2020). Targeting endothelial dysfunction in acute critical illness to reduce organ failure. Anesth. Analg..

[28] Ten Tusscher B., Gudden C., van Vliet S., Smit B., Ince C., Boerma E.C., de Grooth H.S., Elbers P.W.G. (2017). Focus on focus: Lack of coherence between systemic and microvascular indices of oedema formation. Anaesthesiol. Intensive Ther..

[29] Uz Z., Ince C., Shen L., Ergin B., van Gulik T.M. (2021). Real-time observation of microcirculatory leukocytes in patients undergoing major liver resection. Sci. Rep..

[30] Bateman R.M., Sharpe M.D., Singer M., Ellis C.G. (2017). The Effect of sepsis on the erythrocyte. Int. J. Mol. Sci..

[31] Kim J., Lee H., Shin S. (2015). Advances in the measurement of red blood cell deformability: A brief review. J. Cell. Biotechnol..

[32] Bateman R.M., Jagger J.E., Sharpe M.D., Ellsworth M.L., Mehta S., Ellis C.G. (2001). Erythrocyte deformability is a nitric oxide-mediated factor in decreased capillary density during sepsis. Am. J. Physiol. Heart Circ. Physiol..

[33] Donadello K., Piagnerelli M., Reggiori G., Gottin L., Scolletta S., Occhipinti G., Zouaoui Boudjeltia K., Vincent J.L. (2015). Reduced red blood cell deformability over time is associated with a poor outcome in septic patients. Microvasc. Res..

[34] Atasever B., Boer C., Goedhart P., Biervliet J., Seyffert J., Speekenbrink R., Schwarte L., de Mol B., Ince C. (2011). Distinct alterations in sublingual microcirculatory blood flow and hemoglobin oxygenation in on-pump and off-pump coronary artery bypass graft surgery. J. Cardiothorac. Vasc. Anesth..

[35] Atasever B., van der Kuil M., Boer C., Vonk A., Schwarte L., Girbes A.R., Ince C., Beishuizen A., Groeneveld A.B. (2012). Red blood cell transfusion compared with gelatin solution and no infusion after cardiac surgery: Effect on microvascular perfusion, vascular density, hemoglobin, and oxygen saturation. Transfusion.

[36] Yuruk K., Bezemer R., Euser M., Milstein D.M., de Geus H.H., Scholten E.W., de Mol B.A., Ince C. (2012). The effects of conventional extracorporeal circulation versus miniaturized extracorporeal circulation on microcirculation during cardiopulmonary bypass-assisted coronary artery bypass graft surgery. Interact. Cardiovasc. Thorac. Surg..

[37] Vincent J.L., Ince C., Bakker J. (2012). Clinical review: Circulatory shock—An update: A tribute to Professor Max Harry Weil. Crit. Care.

[38] Legrand M., Mik E.G., Balestra G.M., Lutter R., Pirracchio R., Payen D., Ince C. (2010). Fluid resuscitation does not improve renal oxygenation during hemorrhagic shock in rats. Anesthesiology.

[39] Yealy D.M., Kellum J.A., Huang D.T., Barnato A.E., Weissfeld L.A., Pike F., Terndrup T., Wang H.E., Hou P.C., LoVecchio F. (2014). A randomized trial of protocol-based care for early septic shock. N. Engl. J. Med..

[40] Pearse R.M., Harrison D.A., MacDonald N., Gillies M.A., Blunt M., Ackland G., Grocott M.P., Ahern A., Griggs K., Scott R. (2014). Effect of a perioperative, cardiac output-guided hemodynamic therapy algorithm on outcomes following major gastrointestinal surgery: A randomized clinical trial and systematic review. JAMA.

[41] Boyd J.H., Forbes J., Nakada T.A., Walley K.R., Russell J.A. (2011). Fluid resuscitation in septic shock: A positive fluid balance and elevated central venous pressure are associated with increased mortality. Crit. Care Med..

[42] Ince C. (2015). Hemodynamic coherence and the rationale for monitoring the microcirculation. Crit. Care.

[43] Tachon G., Harrois A., Tanaka S., Kato H., Huet O., Pottecher J., Vicaut E., Duranteau J. (2014). Microcirculatory alterations in traumatic hemorrhagic shock. Crit. Care Med..

[44] Bakker J., Ince C. (2020). Monitoring coherence between the macro and microcirculation in septic shock. Curr. Opin. Crit. Care.

[45] Levy M.M., Evans L.E., Rhodes A. (2018). The Surviving Sepsis Campaign Bundle: 2018 update. Intensive Care Med..

[46] De Backer D., Creteur J., Preiser J.C., Dubois M.J., Vincent J.L. (2002). Microvascular blood flow is altered in patients with sepsis. Am. J. Respir. Crit. Care Med..

[47] Yeh Y.C., Chiu C.T. (2019). Association and dissociation of microcirculation and macrocirculation in critically ill patients with shock. J. Emerg. Crit. Care Med..

[48] De Backer D., Donadello K., Sakr Y., Ospina-Tascon G., Salgado D., Scolletta S., Vincent J.L. (2013). Microcirculatory alterations in patients with severe sepsis: Impact of time of assessment and relationship with outcome. Crit. Care Med..

[49] Verdant C.L., De Backer D., Bruhn A., Clausi C.M., Su F., Wang Z., Rodriguez H., Pries A.R., Vincent J.L. (2009). Evaluation of sublingual and gut mucosal microcirculation in sepsis: A quantitative analysis. Crit. Care Med..

[50] Sakr Y., Dubois M.J., De Backer D., Creteur J., Vincent J.L. (2004). Persistent microcirculatory alterations are associated with organ failure and death in patients with septic shock. Crit. Care Med..

[51] Seitz K.P., Qian E.T., Semler M.W. (2022). Intravenous fluid therapy in sepsis. Nutr. Clin. Pract. Off. Publ. Am. Soc. Parenter. Enter. Nutr..

[52] Kanoore Edul V.S., Ince C., Dubin A. (2015). What is microcirculatory shock?. Curr. Opin. Crit. Care.

[53] Kuttab H.I., Lykins J.D., Hughes M.D., Wroblewski K., Keast E.P., Kukoyi O., Kopec J.A., Hall S., Ward M.A. (2019). Evaluation and Predictors of Fluid Resuscitation in Patients With Severe Sepsis and Septic Shock. Crit. Care Med..

[54] Elbers P.W., Wijbenga J., Solinger F., Yilmaz A., van Iterson M., van Dongen E.P., Ince C. (2011). Direct observation of the human microcirculation during cardiopulmonary bypass: Effects of pulsatile perfusion. J. Cardiothorac. Vasc. Anesth..

[55] Dekker N.A.M., Veerhoek D., van Leeuwen A.L.I., Vonk A.B.A., van den Brom C.E., Boer C. (2020). Microvascular Alterations During Cardiac Surgery Using a Heparin or Phosphorylcholine-Coated Circuit. J. Cardiothorac. Vasc. Anesth..

[56] Ball L., Costantino F., Pelosi P. (2016). Postoperative complications of patients undergoing cardiac surgery. Curr. Opin. Crit. Care.

[57] Cherry A.D. (2019). Mitochondrial Dysfunction in Cardiac Surgery. Anesthesiol. Clin..

[58] Uz Z., Aykut G., Massey M., Ince Y., Ergin B., Shen L., Toraman F., van Gulik T.M., Ince C. (2020). Leukocyte-Endothelium Interaction in the Sublingual Microcirculation of Coronary Artery Bypass Grafting Patients. J. Vasc. Res..

[59] Uz Z., Milstein D.M.J., Ince C., de Mol B.A.J.M. (2017). Circulating microaggregates during cardiac surgery precedes postoperative stroke. J. Thromb. Thrombolysis.

[60] Koning N.J., Atasever B., Vonk A.B., Boer C. (2014). Changes in microcirculatory perfusion and oxygenation during cardiac surgery with or without cardiopulmonary bypass. J. Cardiothorac. Vasc. Anesthesia.

[61] Zhu N., Zhang D., Wang W., Li X., Yang B., Song J., Zhao X., Huang B., Shi W., Lu R. (2020). A novel coronavirus from patients with pneumonia in China, 2019. N. Engl. J. Med..

[62] Hamming I., Timens W., Bulthuis M.L., Lely A.T., Navis G., van Goor H. (2004). Tissue distribution of ACE2 protein, the functional receptor for SARS coronavirus. A first step in understanding SARS pathogenesis. J. Pathol..

[63] McGonagle D., O’Donnell J.S., Sharif K., Emery P., Bridgewood C. (2020). Immune mechanisms of pulmonary intravascular coagulopathy in COVID-19 pneumonia. Lancet Rheumatol..

[64] Joffre J., Hellman J., Ince C., Ait-Oufella H. (2020). Endothelial Responses in Sepsis. Am. J. Respir. Crit. Care Med..

[65] Do Espírito Santo D.A., Lemos A.C.B., Miranda C.H. (2020). In vivo demonstration of microvascular thrombosis in severe COVID-19. J. Thromb. Thrombolysis.

[66] Zheng Y.Y., Ma Y.T., Zhang J.Y., Xie X. (2020). COVID-19 and the cardiovascular system. Nat. Rev. Cardiol..

[67] Favaron E., Ince C., Hilty M.P., Ergin B., van der Zee P., Uz Z., Wendel Garcia P.D., Hofmaenner D.A., Acevedo C.T., van Boven W.J. (2021). Capillary Leukocytes, Microaggregates, and the Response to Hypoxemia in the Microcirculation of Coronavirus Disease 2019 Patients. Crit. Care Med..

[68] Brouwer F., Ince C., Pols J., Uz Z., Hilty M.P., Arbous M.S. (2024). The microcirculation in the first days of ICU admission in critically ill COVID-19 patients is influenced by severity of disease. Sci. Rep..

[69] Farquhar I., Martin C.M., Lam C., Potter R., Ellis C.G., Sibbald W.J. (1996). Decreased capillary density in vivo in bowel mucosa of rats with normotensive sepsis. J. Surg. Res..

[70] Abou-Arab O., Beyls C., Khalipha A., Guilbart M., Huette P., Malaquin S., Lecat B., Macq P.Y., Roger P.A., Haye G. (2021). Microvascular flow alterations in critically ill COVID-19 patients: A prospective study. PLoS ONE.

[71] Edul V.S., Enrico C., Laviolle B., Vazquez A.R., Ince C., Dubin A. (2012). Quantitative assessment of the microcirculation in healthy volunteers and in patients with septic shock. Crit. Care Med..

[72] Massey M.J., Hou P.C., Filbin M., Wang H., Ngo L., Huang D.T., Aird W.C., Novack V., Trzeciak S., Yealy D.M. (2018). Microcirculatory perfusion disturbances in septic shock: Results from the ProCESS trial. Crit. Care.

[73] Salgado D.R., Ortiz J.A., Favory R., Creteur J., Vincent J.L., De Backer D. (2010). Microcirculatory abnormalities in patients with severe influenza A (H1N1) infection. Can. J. Anaesth..

[74] Kanoore Edul V.S., Caminos Eguillor J.F., Ferrara G., Estenssoro E., Siles D.S.P., Cesio C.E., Dubin A. (2021). Microcirculation alterations in severe COVID-19 pneumonia. J. Crit. Care.

[75] Kaplan L.J., McPartland K., Santora T.A., Trooskin S.Z. (2001). Start with a subjective assessment of skin temperature to identify hypoperfusion in intensive care unit patients. J. Trauma Acute Care Surg..

[76] Tibby S.M., Hatherill M., Murdoch I.A. (1999). Capillary refill and coreperipheral temperature gap as indicators of haemodynamic status in paediatric intensive care patients. Arch. Dis. Child..

[77] Kruger A., Stewart J., Sahityani R., O’Riordan E., Thompson C., Adler S., Garrick R., Vallance P., Goligorsky M. (2006). Laser Doppler flowmetry detection of endothelial dysfunction in end-stage renal disease patients: Correlation with cardiovascular risk. Kidney Int..

[78] Nichol A.D., Egi M., Pettila V., Bellomo R., French C., Hart G., Davies A., Stachowski E., Reade M.C., Bailey M. (2010). Relative hyperlactatemia and hospital mortality in critically ill patients: A retrospective multi-centre study. Crit. Care.

[79] Garcia-Alvarez M., Marik P., Bellomo R. (2014). Sepsis-associated hyperlactatemia. Crit. Care.

[80] Rathbone E., Fu D. (2024). Quantitative Optical Imaging of Oxygen in Brain Vasculature. J. Phys. Chem. B.

[81] Thomas R., Shin S.S., Balu R. (2023). Applications of near-infrared spectroscopy in neurocritical care. Neurophotonics.

[82] Hanssen H., Streese L., Vilser W. (2022). Retinal vessel diameters and function in cardiovascular risk and disease. Prog. Retin. Eye Res..

[83] Kawasaki R., Cheung N., Wang J.J., Klein R., Klein B.E., Cotch M.F., Sharrett A.R., Shea S., Islam F.A., Wong T.Y. (2009). Retinal vessel diameters and risk of hypertension: The Multiethnic Study of Atherosclerosis. J. Hypertens..

[84] Toraman F., Aksu U. (2015). Monitoring Tissue Oxygenation and Perfusion. Turk. Klin. Anesthesiol. Reanim.-Spec. Top..

[85] Chen M., Knox H.J., Tang Y., Liu W., Nie L., Chan J., Yao J. (2019). Simultaneous photoacoustic imaging of intravascular and tissue oxygenation. Opt. Lett..

[86] Groner W., Winkelman J.W., Harris A.G., Ince C., Bouma G.J., Messmer K., Nadeau R.G. (1999). Orthogonal polarization spectral imaging: A new method for study of the microcirculation. Nat. Med..

[87] Aykut G., Veenstra G., Scorcella C., Ince C., Boerma C. (2015). Cytocam-IDF (incident dark field illumination) imaging for bedside monitoring of the microcirculation. Intensive Care Med. Exp..

[88] Creteur J., De Backer D., Sakr Y., Koch M., Vincent J.L. (2006). Sublingual capnometry tracks microcirculatory changes in septic patients. Intensive Care Med..

[89] Kastelein A.W., Diedrich C.M., de Waal L., Ince C., Roovers J.W.R. (2020). The vaginal microcirculation after prolapse surgery. Neurourol. Urodyn..

[90] De Bruin A.F.J., Tavy A.L.M., van der Sloot K., Smits A., Ince C., Boerma E.C., Noordzij P.G., Boerma D., van Iterson M. (2018). Can sidestream dark field (SDF) imaging identify subtle microvascular changes of the bowel during colorectal surgery?. Tech. Coloproctol..

[91] van Elteren H.A., Ince C., Tibboel D., Reiss I.K., de Jonge R.C. (2015). Cutaneous microcirculation in preterm neonates: Comparison between sidestream dark field (SDF) and incident dark field (IDF) imaging. J. Clin. Monit. Comput..

[92] Boerma E.C., Kaiferová K., Konijn A.J., De Vries J.W., Buter H., Ince C. (2011). Rectal microcirculatory alterations after elective on-pump cardiac surgery. Minerva Anestesiol..

[93] Gilbert-Kawai E., Coppel J., Phillip H., Grocott M., Ince C., Martin D. (2016). Changes in labial capillary density on ascent to and descent from high altitude. F1000Research.

[94] van Zijderveld R., Ince C., Schlingemann R.O. (2014). Orthogonal polarization spectral imaging of conjunctival microcirculation. Graefe’s Arch. Clin. Exp. Ophthalmol..

[95] Pennings F.A., Bouma G.J., Ince C. (2004). Direct observation of the human cerebral microcirculation during aneurysm surgery reveals increased arteriolar contractility. Stroke.

[96] Kastelein A.W., Vos L.M.C., van Baal J.O.A.M., Koning J.J., Hira V.V.V., Nieuwland R., van Driel W.J., Uz Z., van Gulik T.M., van Rheenen J. (2020). Poor perfusion of the microvasculature in peritoneal metastases of ovarian cancer. Clin. Exp. Metastasis.

[97] Hashimoto R., Kurata T., Sekine M., Nakano K., Ohnishi T., Haneishi H. (2018). Two-wavelength oximetry of tissue microcirculation based on sidestream dark-field imaging. J. Biomed. Opt..

[98] Elmoselhi A.B., Shankhwar V., Qaisar R., Hamoudi R., Brix B., Salon A., Goswami N. (2024). Retinal vascular changes and arterial stiffness during 8-month isolation and confinement: The SIRIUS-21 space analog mission. Front. Physiol..

[99] Saloň A., Vladic N., Schmid-Zalaudek K., Steuber B., Hawliczek A., Urevc J., Bergauer A., Pivec V., Shankhwar V., Goswami N. (2023). Sex Variations in Retinal Microcirculation Response to Lower Body Negative Pressure. Biology.

[100] Saloň A., Neshev R., Teraž K., Šimunič B., Peskar M., Marušič U., Pišot S., Šlosar L., Gasparini M., Pišot R. (2023). A pilot study: Exploring the influence of COVID-19 on cardiovascular physiology and retinal microcirculation. Microvasc. Res..

[101] Saloň A., Çiftci G.M., Zubac D., Šimunič B., Pišot R., Narici M., Fredriksen P.M., Nkeh-Chungag B.N., Sourij H., Šerý O. (2023). Retinal venular vessel diameters are smaller during ten days of bed rest. Sci. Rep..

[102] Dinevski D., Lučovnik M., Žebeljan I., Guzelj D., Dinevski I.V., Salon A., De Boever P., Goswami N. (2022). Analysis of Retinal Blood Vessel Diameters in Pregnant Women Practicing Yoga: A Feasibility Study. Healthcare.

[103] Mahdy A., Stradner M., Roessler A., Brix B., Lackner A., Salon A., Goswami N. (2021). A Pilot Study: Hypertension, Endothelial Dysfunction and Retinal Microvasculature in Rheumatic Autoimmune Diseases. J. Clin. Med..

[104] Goswami N., Fredriksen P.M., Lundin K.E.A., Agu C., Elias S.O., Motaung K.S., Brix B., Cvirn G., Sourij H., Stelzl E. (2021). COVID-19 and its effects on endothelium in HIV-positive patients in sub-Saharan Africa: Cardiometabolic risk, thrombosis and vascular function (ENDOCOVID STUDY). BMC Infect. Dis..

[105] De Backer D., Hollenberg S., Boerma C., Goedhart P., Büchele G., Ospina-Tascon G., Dobbe I., Ince C. (2007). How to evaluate the microcirculation: Report of a round table conference. Crit. Care.

[106] Ince C., Boerma E.C., Cecconi M., De Backer D., Shapiro N.I., Duranteau J., Pinsky M.R., Artigas A., Teboul J.L., Reiss I.K.M. (2018). Cardiovascular Dynamics Section of the ESICM. Second consensus on the assessment of sublingual microcirculation in critically ill patients: Results from a task force of the European Society of Intensive Care Medicine. Intensive Care Med..

[107] Hilty M.P., Ince C. (2020). Automated quantification of tissue red blood cell perfusion as a new resuscitation target. Curr. Opin. Crit. Care.

